# Host Density and Competency Determine the Effects of Host Diversity on Trematode Parasite Infection

**DOI:** 10.1371/journal.pone.0105059

**Published:** 2014-08-13

**Authors:** Jeremy M. Wojdak, Robert M. Edman, Jennie A. Wyderko, Sally A. Zemmer, Lisa K. Belden

**Affiliations:** 1 Department of Biology, Radford University, Radford, Virginia, United States of America; 2 Department of Biological Sciences, Virginia Tech, Blacksburg, Virginia, United States of America; Technion-Israel Institute of Technology Haifa 32000 Israel, Israel

## Abstract

Variation in host species composition can dramatically alter parasite transmission in natural communities. Whether diverse host communities dilute or amplify parasite transmission is thought to depend critically on species traits, particularly on how hosts affect each other’s densities, and their relative competency as hosts. Here we studied a community of potential hosts and/or decoys (i.e. non-competent hosts) for two trematode parasite species, *Echinostoma trivolvis* and *Ribeiroia ondatrae*, which commonly infect wildlife across North America. We manipulated the density of a focal host (green frog tadpoles, *Rana clamitans*), in concert with manipulating the diversity of alternative species, to simulate communities where alternative species either (1) replace the focal host species so that the total number of individuals remains constant (substitution) or (2) add to total host density (addition). For *E. trivolvis*, we found that total parasite transmission remained roughly equal (or perhaps decreased slightly) when alternative species replaced focal host individuals, but parasite transmission was higher when alternative species were added to a community without replacing focal host individuals. Given the alternative species were roughly equal in competency, these results are consistent with current theory. Remarkably, both total tadpole and *per-capita* tadpole infection intensity by *E. trivolvis* increased with increasing intraspecific host density. For *R. ondatrae*, alternative species did not function as effective decoys or hosts for parasite infective stages, and the diversity and density treatments did not produce clear changes in parasite transmission, although high tank to tank variation in *R. ondatrae* infection could have obscured patterns.

## Introduction

**Figure 1:**
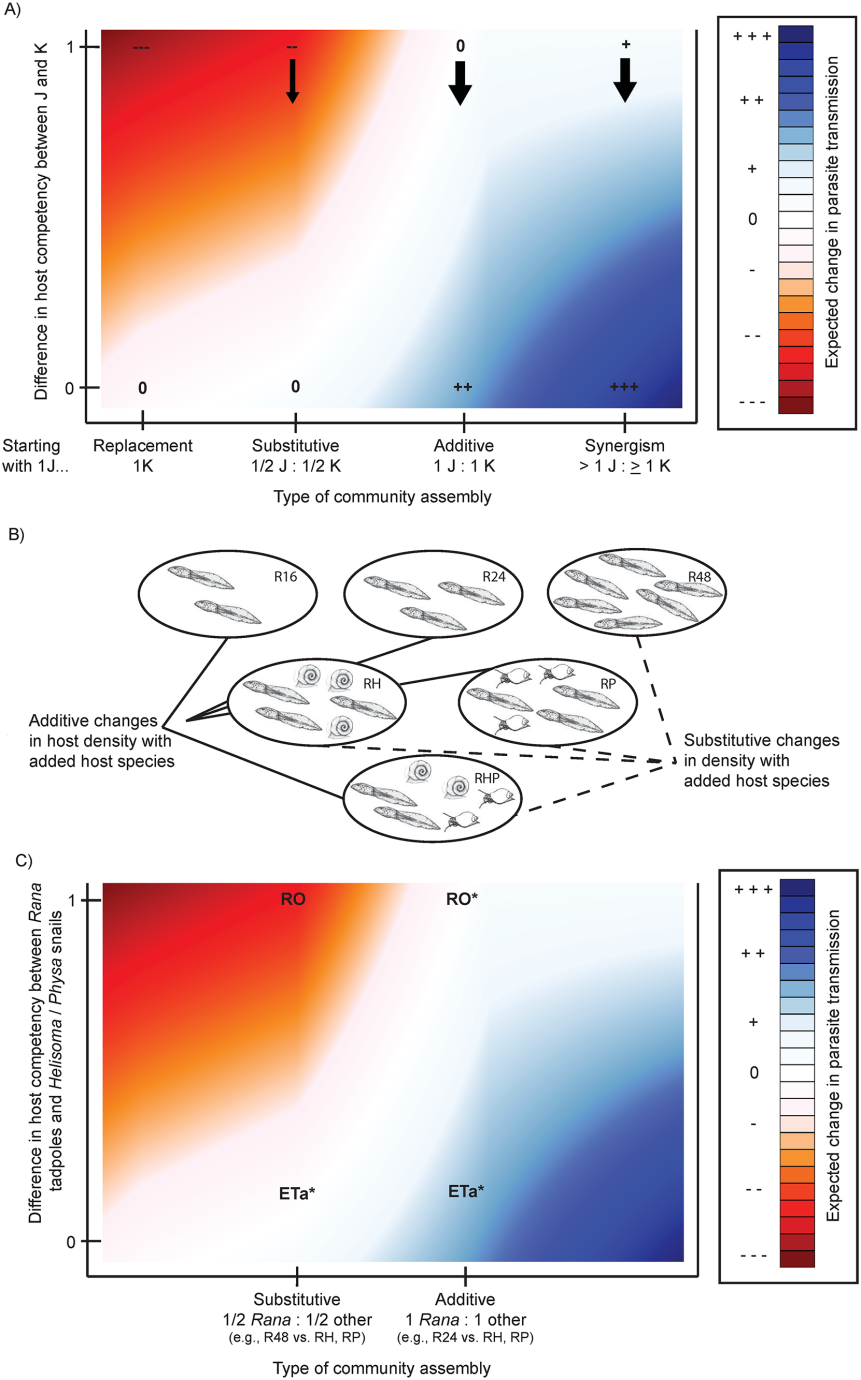
A conceptual model of the effects of hosts’ density and competency on parasite transmission, our experimental design depicted graphically, and some predictions. **A)** If we imagine starting with a single host species *J*, and add to it a second host species *K*, the horizontal axis describes the range of possibilities in terms of their eventual densities. For example, resource competition or predator-prey interactions might lead to *K* extirpating *J* (“replacement”), or coexisting at reduced densities (“substitutive”). If *J* and *K* are largely independent, the introduction of *K* might leave the density of *J* unchanged (“additive”). If *J* and *K* are mutualists directly or indirectly, *J* may increase in abundance after the introduction of *K* (“synergism”). The y-axis describes the range of potential differences in host competency between species *J* and *K*. The graphical space thus represents all possible combinations of density effects and differences in host competency. The color indicates expected changes (based on qualitative, conceptual understanding) to total parasite transmission, given the type of community assembly and host competency. Black arrows represent situations where species *K* could have particularly strong effects on infection in species *J* through encounter reduction, that is, by absorbing parasites that otherwise would have intercepted species *J* individuals. **B)** *Rana* tadpole and snail host/decoy density in each treatment. Comparing R16, R24, and R48 tests the effects of tapdole density. Comparing R16 vs. RHP, and R24 vs. RH or RP, tests for host diversity effects in communities that assemble additively. Comparing R48 vs. RHP or RH or RP tests for host diversity effects in communities that assemble substitutively. **C)** The graphical space and background color are as before in panel A, but now superimposed are the experimental situations created: substitutive and additive community assembly for RO (where alternative species are incompetent hosts), and for ETa (where alternative species are fairly competent hosts). This graphical approach gives us clear *a priori* expectations for the results of the experimental design. Asterisks indicate where experimental results coincide with these expectations.

The composition of the ecological community in which a parasite species and its hosts are embedded can dramatically influence transmission dynamics, but there is still debate about the balance among mechanisms that determine transmission. Recent studies suggest that how host species assemble or disassemble in a community, and more specifically, how the members of a diverse community differ in abundance from the members of a less diverse community, are critical in determining how parasite transmission will change following biodiversity loss (Figure 1A, horizontal axis; [1]–[4]). Thus, understanding the impact of changing host densities is an important step in being able to understand the expected impacts of biodiversity loss on parasite dynamics. On one hand, the presence of a more diverse host species community may reduce the population sizes of some of the hosts (“substitution”), especially if the hosts are functionally similar (i.e. at the same trophic level) or if one host preys upon the other. Hall et al. [5] provided evidence for resource competition among several species of *Daphnia* that act as hosts (of varying competency) for a fungal pathogen in lakes, and reported negative correlations in abundance of the host species in the field. Similarly, some of the amphibian hosts (e.g., *Ambystoma* salamanders, newts) of trematodes may prey on other amphibian hosts (anuran larvae), reducing their density [6]. Functionally similar non-host species may also change the density of a host species through resource competition or other mechanisms [7], [8] – i.e. susceptible host regulation *sensu* [9].

However, if hosts are unlikely to require the same resources or share predators, or are mutualists, we might expect the presence of more host species to equate to more total host individuals (“additivity” or even “synergism”, Figure 1A). For example, *Fasciola hepatica* Linnaeus 1758 is a trematode that infects livestock, wild ruminants, rodents, and humans as definitive hosts [10], [11]. Many of these species are only weakly connected in food webs, and thus changes to community composition of these hosts may result in additive changes in host density. Similarly, *Gongylonema pulchrum* Molin 1857 is a nematode that can use a very wide array of species as definitive hosts, including monkeys, bears, rabbits, rodents, deer, sheep, cattle, and pigs [12]. Surprisingly, even the abundance of species that are putative competitors may be largely independent, as appears to be the case for the granivorous/omnivorous rodents that serve as key hosts for the Lyme Disease bacterium in the Northeastern USA [13].

Transmission must be a product of both the density and competency of each host, and a reduction of parasite transmission in systems with high host diversity (i.e., a dilution effect) depends on variation in host competency (vertical axis of Figure 1A; [3], [14]–[16]). In general, high competency hosts may be the most likely species to persist in disturbed, low diversity habitats, if there is a trade-off between “weedy” life history traits that favor persistence and decreased immune function that readily permits infection [17]–[22] (but see [23]–[25]). Moreover, differences in host species composition can impact parasite transmission via encounter reduction ([9]). Some species may absorb parasite infective stages, whether or not they themselves get infected, reducing encounters between the most competent hosts and parasites. Encounter reduction should have the strongest effect when the species intercepting parasites have low or no competency (Figure 1A, black arrows). If common, these trade-offs would suggest increasing diversity should routinely be correlated with decreasing community competence. Johnson et al. [26] demonstrated that among a large set of ponds in California, the amphibian species that serves as the most competent second intermediate host for *Ribeiroia ondatrae* Looss 1907 tends to persist in ponds across the species diversity gradient, but exists at lower densities when many other species are present. Correspondingly, parasite infection across all host species was highest in low diversity communities dominated by the most competent host. Similar patterns have been seen in viral infection dynamics in California grassland systems, with non-random community disassembly contributing to dominance of highly-competent viral hosts [27]. Whether it turns out, across studies of diverse systems and taxa, that low diversity host communities are often nested subsets that contain the most competent hosts from higher diversity communities awaits further investigation [3], [16].

However, the simple linear increase in contact rate with increasing host density assumed by most disease models (and our predictions in Figure 1A) may not be appropriate for some disease systems, potentially including trematodes [28]–[31]. Predicting how total transmission among all hosts should change with host diversity may be less clear if contact rate is a non-linear function of host density [4]. For example, some trematodes release more cercariae infective stages when their current first intermediate hosts are in the presence of more second intermediate hosts [32], [33]. Also, if parasite infective stages are more or less uniformly distributed in space, some may never be near enough to a host to cause an infection – but with greater host abundance, more infective stages will be near hosts, and per-capita infection rates may not decline with greater host abundance; the additional hosts just intercept parasites that otherwise would have been “wasted”. Lastly, the infection success of individual parasites may decrease at high ratios of parasites to hosts [34], and if so, more abundant hosts would reduce the ratio of parasite to hosts, and increases parasite success rate.

Here we simultaneously studied the trematodes *Echinostoma trivolvis* Rudolphi 1809 and *Ribeiroia ondatrae*, both common parasites in the U.S. that can use amphibians as second intermediate hosts and that can induce pathology in their hosts [35]–[40]. To parse out the effects of host density and diversity on parasite transmission, we created treatments simulating both additive and substitutive changes in host community composition with increasing diversity. We built on prior work, by using a suite of alternative species (snails) that were at extreme ends of the spectrum for host competency; for *E. trivolvis*, our alternative snail hosts were roughly equivalent in competence compared to the focal tadpole host, and for *R. ondatrae* the alternative snail species were potential decoys but not competent hosts. Moreover, we focus not just on transmission to the focal host, but on community-wide transmission to all of the potential species, that is, the trematode component population found in all second intermediate hosts. We found that for *E. trivolvis*, total parasite transmission among all host species was higher when hosts assembled additively, but remained essentially constant when hosts assembled substitutively. For *R. ondatrae*, there were no clear changes in transmission in response to the presence of other species or to changes in focal host density. We therefore observed that transmission was a complex function of host diversity, density, and competence, yet predictable based on existing theory.

## Materials and Methods

### Study system

Digenetic trematodes are a diverse group of parasitic flatworms that infect all vertebrate classes, but for the majority of species, little is known. However, both *E. trivolvis* (hereafter ET) and *R. ondatrae* (hereafter RO), the trematode responsible for many frog limb malformations [35], [36], have been well-studied and have become models for understanding ecological effects in host-parasite systems [41]–[43]. Both ET and RO infect three successive hosts. Sexual reproduction occurs within the definitive host, and eggs exit the definitive host in feces. Eggs that fall into water hatch into short-lived, free-swimming miracidia. Miracidia penetrate and infect pond snails as first-intermediate hosts. Within infected snails, the trematode undergoes an embryonic amplification via multiple intramolluscan stages (i.e. sporocysts/rediae) that results in the production of thousands of free-living cercariae. Cercariae emerge from the snail, and then seek, penetrate and form cysts (metacercariae) in second intermediate hosts (e.g., tadpoles or fish for RO; tadpoles, snails, or other invertebrates for ET). The life cycle is completed when an infected second intermediate host is ingested by a definitive host, in which the parasite excysts and develops into an adult worm. RO adults are found mainly in herons and raptors [36]. While ET was historically thought to have a wide variety of definitive mammalian and avian hosts [44], molecular analysis suggests there are cryptic lineages within the genus *Echinostoma* in North America [45], and the definitive host specificity within each lineage has yet to be determined. In these experiments, we used *E. trivolvis* lineage *a* (hereafter “ETa”; [45]), which uses *Helisoma trivolvis* Say 1817 snails as its first intermediate host (as does RO).

Trematodes represent a broad class of wildlife parasites with complex life cycles involving multiple host or vector species that operate at different spatial scales. We used ET and RO because both species can infect and cause pathology in amphibians and they often co-occur in nature [6], [46]. In addition, they provide examples of broader (ET) and narrower (RO) second intermediate host specificity in our experiments.

### Experimental design

Our main objective was to examine the effects of host diversity and community composition on parasitism by two larval trematode species that differ in second intermediate host specificity. We manipulated second intermediate host diversity, and density of a focal host (larval green frogs, *Rana clamitans* Latreile 1801*)*, with six treatments: 16, 24 or 48 *Rana* tadpoles alone (R16, R24, R48), 24 *Rana* tadpoles plus 24 *Helisoma* snails (RH), 24 *Rana* tadpoles plus 24 *Physa gyrina* Say 1821 (hereafter *Physa*) snails (RP) and a final three species treatment with 16 *Rana* tadpoles, 16 *Physa* snails and 16 *Helisoma* snails (RHP) (Figure 1B). For ETa, the snail species represented viable alternative hosts. For RO, which does not infect snails as second-intermediate hosts, the snail species represented potential decoys, rather than alternative competent hosts, for cercariae. Trematode cercariae have often been noted to penetrate dead-end or decoy species (see [47] for a review), even including plants [48].

By comparing treatments R24 with RH or RP, we could assess host diversity effects for ETa infection if species assemble additively (that is, when having more species also means having more total individuals – Figure 1B). By comparing R48 with RH, RP, or RHP, we could assess host diversity effects for ETa infection if species assemble substitutively. In contrast, for RO our treatments simulated changes in the presence of decoys with or without compensatory changes in focal host abundance. Each treatment was replicated six times for a total of 36 mesocosms.

Mesocosms were 1000 L outdoor cattle-watering tanks (bottom area of 2.0 m^2^) placed at Virginia Tech’s Kentland Farm, Montgomery County, Virginia. Tanks were filled with well water and 200 g of dry, mixed-hardwood leaf litter was added to each tank (9 Jul 2010). Shade-cloth lids were affixed over the tanks to discourage entry or exit of organisms.

Parts of three *Rana* egg masses were collected from a pond near the tanks (16 and 18 Jun 2010) and reared in a separate tank until the experiment began. The three potential second intermediate host species, *Rana*, *Helisoma* and *Physa,* were all added to the tanks on 28 Jul 2010. Tadpoles averaged 96 mg wet weight (±57; 1 SD) and were mostly Gosner stage 25 (range 25–27; [49]). *Helisoma* snails initially averaged 9.8±1.73 mm shell diameter and 9.9±4.4 mg shell-less dry weight, while *Physa* snails initially averaged 6.2±1.6 mm shell height and 2.2±1.9 mg shell-less dry weight (mean±1 SD for each measurement). These snails were raised in outdoor mesocosms from eggs and thus had no possibility of being infected by trematodes before the experiment started.

*Helisoma* snails (17.5±3.0 (1 SD) mm shell diameter) that were naturally infected as first-intermediate hosts of either ETa or RO and were actively shedding cercariae were collected from a different pond in Montgomery County, Virginia, for use as sources of cercariae in the tanks. To reduce total parasite pressure on the host communities in the tanks, only half of the cattle tanks had an infected snail on any given day, which meant that we used a total of 18 infected first-intermediate host snails in the experiment. Five of the eighteen snails were infected with RO, and the remaining 13 were infected with ET. This ratio is close to the ratio of relative abundance of the two parasites at our collection site at the time of the experiment (72% ET: 28% RO in experimental tanks, 62% ET: 38% RO in local survey; LKB *unpublished data*).

Infected, shedding snails were suspended in tanks in fiberglass window screen cages (cylinders with 1 mm mesh size, 10 cm diameter, extending 40 cm deep and 10 cm above the water’s surface). Infected snail cages were added on 29 Jul 2010, the day after hosts were added, and they were subsequently rotated randomly each day from tank to tank across treatments (and with all the tanks receiving an empty, no parasite bag every other day). This rotation served to introduce ETa and RO into every tank, and to account for variation in shedding rates among individual snails. Thus, all tanks received cercariae from many different snails over the course of the experiment, and all tanks experienced ∼0.5 infected snails per day on average (0.25 infected snails/m^2^ per day; similar to field densities of first intermediate trematode host densities in local ponds: range of 0.008–1.74 infected snails/m^2^, average 0.5 infected snails/m^2^; S. Hopkins and J. Wojdak, *unpublished data*). Temporal heterogeneity in parasite presence is also realistic, in that both snails infected as first intermediate hosts and potential second intermediate hosts are moving in time and space.

The experiment ended when infected snails were removed on 25 Aug 2010, a total duration of 27 days. All animals were collected over the next three days. Tadpoles were euthanized with buffered MS-222 and developmental stage was determined according to [49]. Tadpoles and snails were weighed, and then were frozen until dissection. Dissections were conducted blind with respect to treatment, and metacercariae were enumerated (*as in* [6]). This study was carried out in accordance with the recommendations in the Guide for the Care and Use of Laboratory Animals of the National Institutes of Health, and was approved by the Institutional Animal Care and Use Committee at Virginia Tech (Protocol Number: BIOL-09-107). Vertebrate animals (*Rana clamitans* egg masses) were collected from a private pond on Virginia Tech property, and they are not a threatened or endangered species.

### Statistical Methods

Parasites are often aggregated among hosts, with many individuals having little or no infection, and some being highly infected (negative binomial distribution; [50]). Because we had counts of parasites for each individual from each tank, we used tank as a random factor to account for the correlation among individuals in a tank – creating NB generalized linear mixed models (NB GLMMs) with log links. Data exploration before the analyses suggested possible zero-inflation, that is, excess zeroes compared to a standard NB distribution [51]. For each parasite species and host combination we determined whether including zero-inflation improved the fit of the model; it did not improve the fit for ETa infection in tadpoles or snails, but it did improve the fit for RO infection in tadpoles.

Models for the number of metacercariae in *Rana* tadpoles (infection intensity) contained categorical predictors for the presence/absence of each alternative host/decoy species (*Physa* and *Helisoma*) and an interaction term (*Physa* * *Helisoma*), tadpole density (16, 24, or 48) and individual tadpole mass, and the potential mass by density interaction. In the model for RO infection in tadpoles, we also included a zero-inflation term, as mentioned above, and included the count of ETa metacercariae as a potential covariate because ETa metacercariae turned out to be much more abundant than RO. Infection by ETa in *Helisoma* and *Physa* snails was analyzed separately with NB GLMMs with log links, with only a single treatment term comparing the RH or RP treatment, respectively, with the RHP treatment. It is important to note, though, that intraspecific snail density and the presence of the other snail species are confounded in this comparison by the nature of the experimental design; adding density controls for all three host species was not logistically feasible.

We used Bayesian approaches with Markov chain Monte Carlo (MCMC) methods and uninformative priors (*following* [52], [53]) to fit the infection intensity models. We used both Bayesian approaches (here) and frequentist approaches (later) as a matter of pragmatism [54], that is, to be able to work with the complexity of zero-inflated negative binomial generalized linear mixed models. Analyses in WinBUGS used three MCMC chains with random starting values for the effect of tank and initial parameter values generated from similarly structured models run in the package glmmADMB (which uses non-linear optimization, and at the time of analysis was still in beta-testing). Most models were run for 200,000 draws from the posterior distribution, with a thinning rate of 200, and a burn-in period of 100,000 draws, resulting in a final sample of 1500 draws from the posterior distributions. The ZINB GLMM for RO infection in tadpoles required 1,000,000 draws, a thinning rate of 500, and a burn-in period of 500,000 draws, resulting in a final sample of 1000 draws from the posterior distribution.

Analyses from WinBUGS were assessed for convergence visually and by using the Gelman-Rubin statistic [55]. Models were validated by plotting Pearson residuals against predicted values and against each covariate, and by comparing predicted values to observed values. Mean parameter values and 95% Bayesian credible intervals (BCIs) were calculated from the posterior probability density of model parameters, and are often quite similar to frequentist confidence intervals found from maximum likelihood model fits. To draw inferences from these models we compared 95% BCIs with zero – covariates with parameters whose CIs did not overlap zero were considered influential. Parameter estimates from these infection models can be back-transformed by exponentiation (e.g., *e^x^* where *e* is the base of the natural log, and *x* is the parameter estimate).

Tadpole survival and the prevalence of tadpole infection by ETa and RO were analyzed with binomial generalized linear models (GLMs) using logit links. These models included categorical predictors for the presence/absence of each alternative host/decoy species (*Physa* and *Helisoma*) and an interaction term (*Physa* * *Helisoma*), and a term for tadpole density (16, 24, or 48), all as fixed factors. The models also included an inflation factor to account for overdispersion (“quasi-likelihood” approach; [56]). Survival of *Helisoma* and *Physa* snails was analyzed separately with binomial generalized linear models (GLMs) with logit links, with only a single treatment term comparing the RH or RP treatments, respectively, with the RHP treatment. Parameter estimates from the models with logit links can be back-transformed by the function 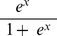 where *e* is the base of the natural log and *x* is the parameter estimate.

The (log) mass of individuals of each host species (*Rana*, *Helisoma*, *Physa*) was analyzed separately with a treatment structure similar to those above for survival, but with Gaussian (normal) linear mixed models with identity links, using tank as a random term to account for the dependence of individuals from the same tank. All of the models assessing mass, infection prevalence, and survival were validated by plotting Pearson residuals against predicted values and against each covariate, by comparing predicted values to observed values, and by assessing overdispersion.

We used R version 2.15.0 [57] including packages MASS [58], glmmADMB [59], and R2WinBUGS [60], and WinBUGS version 1.4.3 software [61].

## Results

### Infection intensity among tadpoles

**Figure 2:**
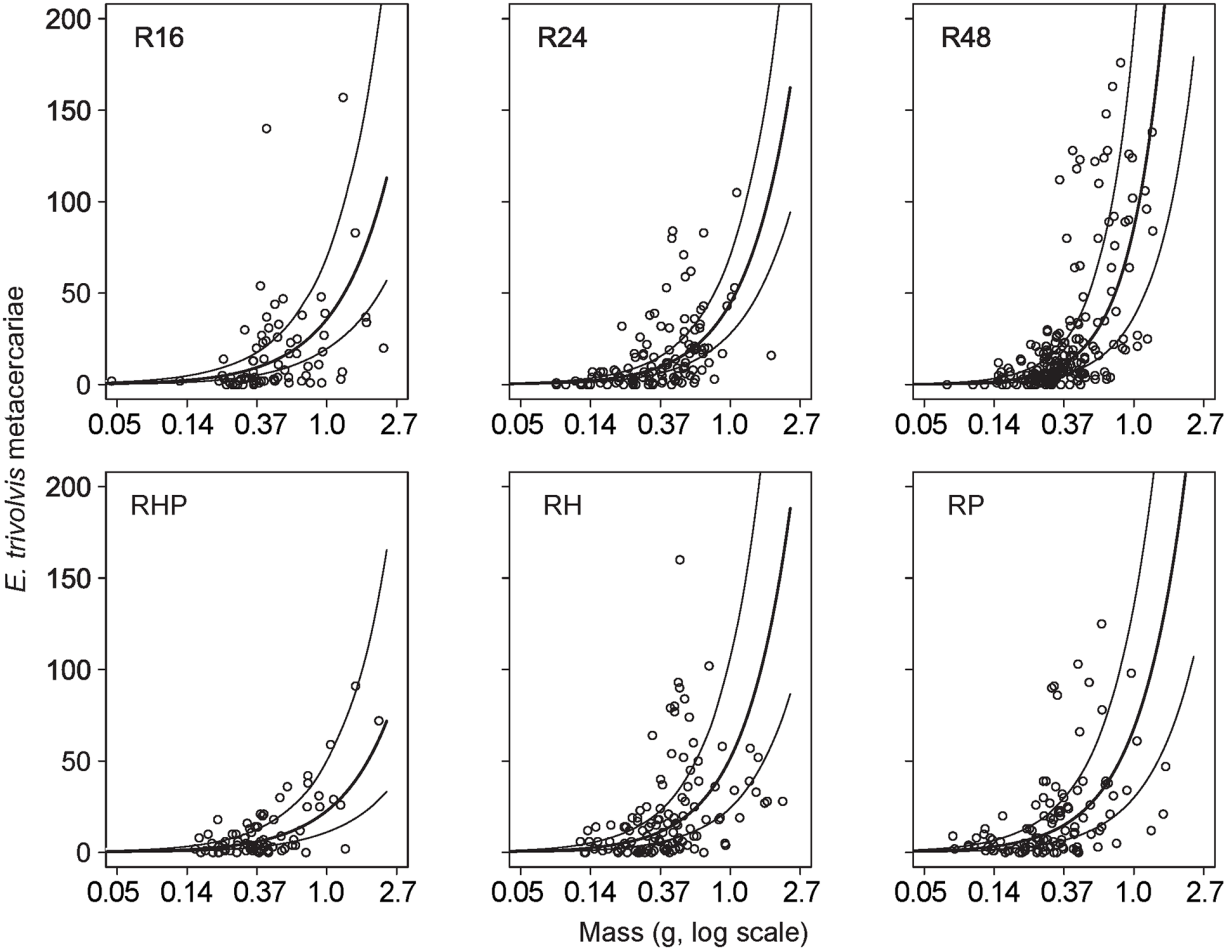
Counts of *E. trivolvis* metacerariae versus tadpole mass (log scaled), for the six tadpole density and host diversity treatments. Dots are observed infection intensities in individual tadpoles, thick lines are the predicted (from NB GLMM model) infection intensity for an animal of a given mass in that treatment, and thin lines represent 95% Bayesian credible intervals. Larger animals were more likely to be infected, but the rate of increased infection with size depended on treatment. Note that per-capita ETa infection increased with *Rana* density (R16 vs. R24 vs. R48), but was not affected by the addition of alternative hosts (RHP vs. R16, RH & RP vs. R24).

**Figure 3:**
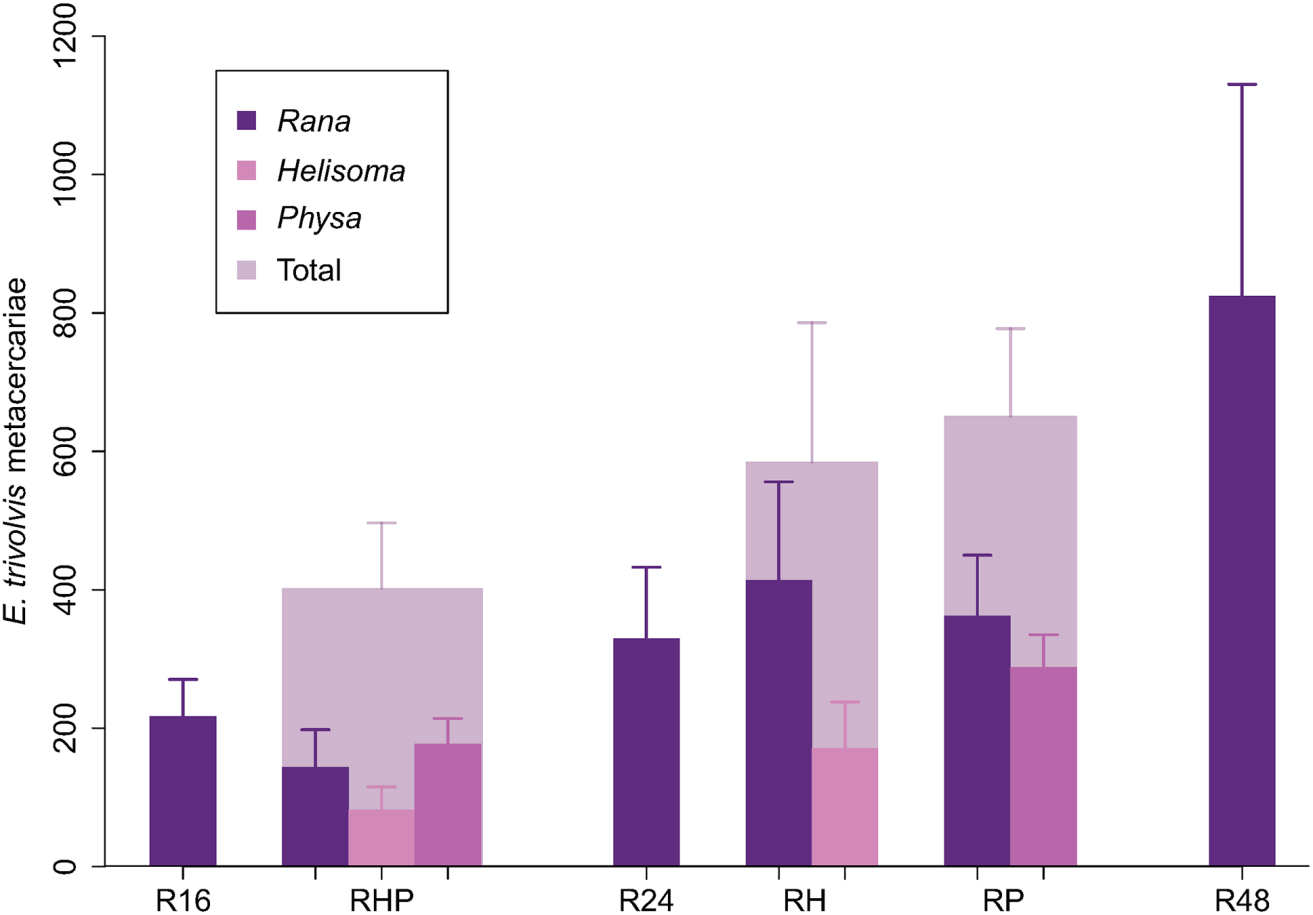
*E. trivolvis* infection by host species, and in total, for each treatment. Means plus one standard error are reported. Shades of purple represent the individual host taxa, while the wider gray bars represent the total of multi-species communities. Total infection increased with *Rana* density (R16 vs. R24 vs. R48). There was more total infection in diverse communities when host densities were additive (R16 vs. RHP or R24 vs. RH/RP), but equal infection in diverse communities when host densities were substitutive (RHP/RH/RP vs. R48).

The number of ETa metacercariae in *Rana* tadpoles depended on *Rana* density and tadpole mass interactively (Table S1; density parameter = 0.027, 95% BCI (−0.011, 0.061); log(mass) parameter = 0.996, 95% BCI (0.649, 1.369); interaction = 0.021, 95% BCI (0.009, 0.033)) – larger tadpoles had more metacercariae generally, but that pattern was stronger at higher tadpole densities (Figure 2). The presence of *Helisoma* had no discernible effect on the number of ETa metacercariae in tadpoles (Table S1; parameter = 0.154, 95% BCI (−0.692, 0.992); Figure 3 – bars for *Rana* in R24 vs. RH), nor did the presence of *Physa* (parameter = 0.412, 95% BCI (−0.458, 1.257); Figure 3 – bars for *Rana* in R24 vs. RP), nor did their joint presence (parameter = −1.030, 95% CI (−2.401, 0.300); Figure 3 – bars for *Rana* in R16 and RHP). The aggregation parameter (alternatively called the size or dispersion parameter, *k*) for the negative binomial distribution of ETa infection intensity in tadpoles was 1.591 (95% BCI (1.398, 1.808)); as *k* approaches infinity a negative binomial distribution approaches the Poisson distribution, and as *k* approaches 0 the counts of parasites are maximally aggregated among hosts. Noticeably, very few tadpoles below 0.37 g (−1 on natural log scale) wet mass had >25 parasites (Figure 2).

**Figure 4:**
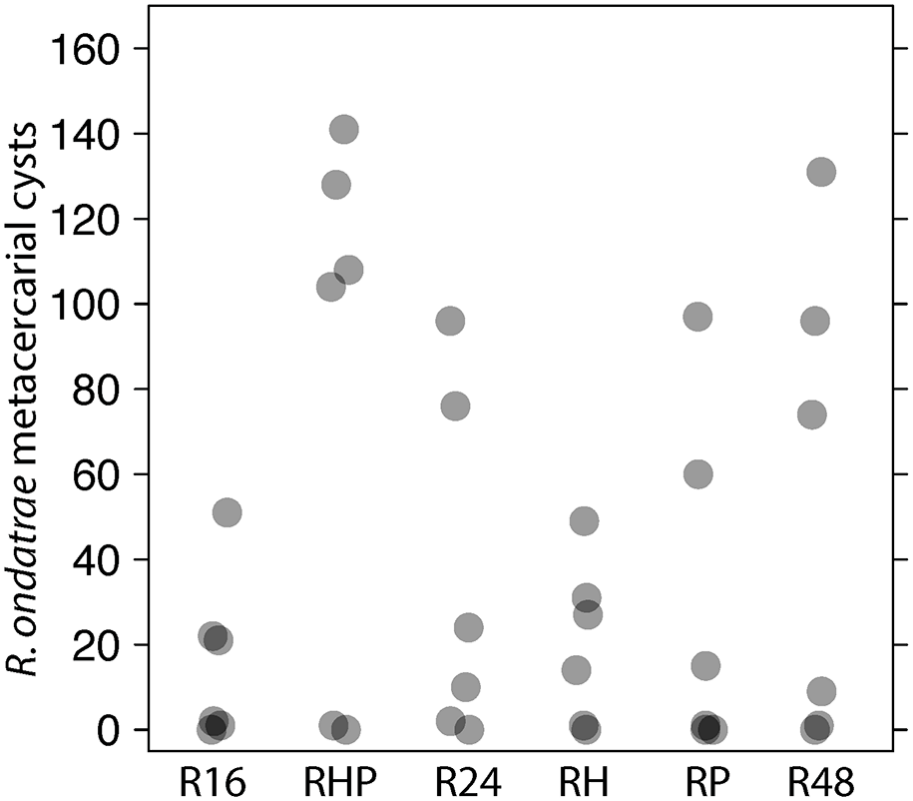
*R. ondatrae* infection in each of the six tadpole density and host/decoy treatments. Each point represents the total number of metacercariae among all the animals in a single tank. The high tank to tank variability within each treatment made detecting any potential treatment differences difficult.

RO infection intensity was relatively low compared to ET, with a mean intensity of only 4.65 metacercariae per infected tadpole, while ETa averaged 21.7 metacercariae per infected tadpole. RO infection did not depend strongly on snail decoy or *Rana* density treatments or tadpole mass (Figure 4, Table S1: all 95% credible intervals for those parameters overlapped zero), but great tank to tank variability (evident in magnitude of the estimated “tank” factor parameter in Table S1) may have contributed to an inability to detect treatment differences. RO infection was negatively related to ETa infection intensity (parameter = −0.008, 95% BCI (−0.013, −0.003)). The aggregation parameter (*k*) for the negative binomial model for RO infection in tadpoles was 1.124 (95% BCI (0.849, 1.929)). The zero-inflation parameter was 0.108 (95% BCI (<0.001, 0.153)), suggesting slightly more zeroes (i.e., uninfected tadpoles) were observed than expected from the negative binomial distribution.

### Infection intensity among snails

Infection intensity of ETa in *Helisoma* did not differ markedly among host species/density treatments or among snails of different mass (Table S1; NB GLMM, 95% BCIs for all parameters overlapped zero), and infection intensity of ETa in *Physa* only depended strongly on snail mass, with more metacercariae in larger snails (Table S1; NB GLMM, log (mass) parameter = 1.266, 95% BCI (0.648, 2.024)). The aggregation parameters for the negative binomial distributions of ETa in *Helisoma* and *Physa* were 0.565 (95% BCI (0.443, 0.723)), and 1.442 (95% BCI (1.120, 1.812)), respectively, indicating greater aggregation among *Helisoma*.

### Total parasite transmission among all hosts

Community-wide ETa metacercarial infection (i.e. among all host species) depended on *Rana* density (Table S1); in general, there were more cysts in communities with more tadpoles (Figure 3; R16 vs. R24 vs. R48; parameter = 0.042, 95% BCI (0.017, 0.070)). Increasing total host density by adding either snail host resulted in an increase in total infection (Figure 3; RH vs R24 - *Helisoma* parameter = 0.627, 95% BCI (−0.067, 1.348); RP vs. R24 - *Physa* parameter = 0.731, 95% BCI (0.128, 1.444); custom contrast between R16 and RHP = 0.926 (95%BCI (0.023, 1.848)). Having both snail species present resulted in roughly equivalent total infection as having just one snail species present (Figure 3; RHP vs. RH/RP - interaction parameter = −0.774, 95% BCI (−1.862, 0.291)). There was a trend towards decreasing total infection intensity as the focal host was partially replaced by other host species (i.e. R48 vs. RH, RP, RHP – custom contrasts computed from posterior parameter distributions). Though for all of these contrasts the BCIs overlapped zero, based on the posterior distribution for the comparison between R48 and RHP, for instance, there was an 85% probability of a decrease in parasitism with increasing diversity (a negative value for that parameter). There was presumably low power to discern differences from treatment R48 because of its large variance, though this large variance is expected because for negative binomially distributed data the variance increases with the mean, and R48 had the largest mean. The aggregation parameter (*k*) for the negative binomial distribution of total ETa in tanks was 2.038 (95% BCI (1.221, 3.056)).

### Infection prevalence among hosts

There were no significant effects of *Rana* tadpole density or host/decoy diversity on the prevalence of ETa or RO infection in tadpoles (i.e. proportion of individuals infected; binomial GLMs; Table S2). Overall, 91% of tadpoles were infected with ETa, and 43% were infected with RO. The prevalence of ETa infection in *Helisoma* did not differ between RH and RHP treatments (binomial GLM; Table S2; 69% overall), nor did the prevalence of ETa infection in *Physa* differ between RP and RHP treatments (binomial GLM; Table S2; 95% overall).

### Host survival and mass

Tadpole survival did not depend on *Rana* tadpole density or host diversity (binomial GLM; Table S2). Overall, 76% of tadpoles survived the experiment, thus a non-trivial proportion of the total larval trematodes that entered hosts may not have been accounted for because of host mortality. Tadpole mass did not differ with intraspecific density treatments or in the presence of *Helisoma* snails (Gaussian GLMM; Table S2), and only showed a marginal negative response to the presence of *Physa* snails (parameter (SE) = −0.24 (0.13), t = −1.92, p = 0.064). By the end of the experiment, the median tadpole mass was 0.366 g (±0.30; 1 SD), and the median Gosner stage was 27 (±1.4; 1 SD). There were no significant differences in snail mass (Gaussian GLMMs; Table S2) or survival (binomial GLMs; Table S2) between treatments with snails (i.e. RH vs. RHP, RP vs. RHP). Overall, 92% of *Helisoma* and 79% of *Physa* survived the experiment.

## Discussion

As we consider how to interpret the results found in our experiment, it may be profitable to start with some basic expectations. Figure 1A shows the range of expected outcomes, in terms of changes to parasite transmission, resulting from adding a host species to a system already containing a single, most competent host species. In general, when additional hosts are less competent or cause reductions in the abundance of the primary host, we expect a reduction in transmission (as described many times in discussions of the dilution effect [14], [26], [27].

While including two trematode species with different second intermediate host specificity complicated our experiment, it represents a more realistic host-parasite community and provides an interesting point of comparison. *Rana*, *Helisoma*, and *Physa* are all reasonably good second intermediate hosts for ETa [40], [62], [63]. Thus, for ETa we expected increasing total community infection with additive changes in host communities, and no change in infection (or a slight decrease due to small differences in competency) with substitutive changes in host communities (Figure 1C). Notably we found results concordant with these predictions: total community infection by ETa among all hosts increased when snails were “added” to tadpoles (R16 vs. RH, RP, or RHP; Figure 3), and was about the same or somewhat less when snails “replaced” tadpoles (RHP, RH, or RP vs. R48; Figure 3, grey bars).

For RO, snails were not competent hosts, and adding them to the community was expected to change infection little, *unless* they 1) reduced encounter rates between RO and *Rana* tadpoles by absorbing cercariae (i.e. acting as decoys), 2) altered tadpole behavior, or 3) altered tadpole survival through resource competition (i.e. susceptible host regulation *sensu* [9]). Previous experiments with trematode cercariae and miracidia (another free-swimming larval stage that needs to find hosts) suggest many taxa can serve as effective decoys [47], distracting larval trematodes from finding and infecting their primary target species. We found that total RO infections were insensitive to changes in snail species presence. However, the high tank-to-tank variability in RO infection success, even within treatments, tempers our ability to confidently declare a lack of response to treatments.

Now, if we consider these expectations and main results, we can start to see how difficult it will be to *quantitatively* predict changes in transmission with changes in real host communities. For example, the dilution effect can be driven by encounter reduction [9], [64] - a numerical reduction in available infective agents because alternative hosts or decoys absorb these infective stages before a more competent host can become infected. Here, the per-capita infection of *Rana* tadpoles by ETa was not changed by adding alternative hosts (e.g., comparing “*Rana*” bars in R16 vs. RHP or R24 vs. RH/RP in Figure 3). Our results suggest that even when alternative hosts are in fact intercepting parasites they still may *not necessarily* reduce total infection for a focal host.

These surprising results may be a function of the high density and limited mobility of these parasites, and their general low likelihood of successfully infecting a host. For example, if one imagines a large habitat with parasites of limited mobility initially uniformly distributed in space, then in many areas of the habitat the parasites have no chance of finding a host when host density is low. Adding more host individuals, that are also distributed in space, puts more parasites within “reach” of finding a host, at least until some host density at which all parasites can find hosts. In our experiment, cercariae all emerged from a single point source in the middle of the tanks (caged, infected snails), and presumably slowly dispersed in search of a host.

Johnson et al. [65] found a reduction in total infection of RO when non-competent *Hyla* treefrog tadpoles were experimentally substituted for competent *Bufo* toad tadpoles. Total community infection was not reported in that study when competent *Rana* tadpoles were substituted for half of the *Bufo* tadpoles, but given that total infection among *Bufo* tadpoles was unchanged, and *Rana* tadpoles presumably carried some cysts as they are susceptible to RO, total community-wide infection probably increased with diversity in that experiment. Thus, even among very similar disease systems, the outcomes of altered host diversity and abundances will depend critically on individual species traits. The good news is that these superficially disparate results – reduced, unchanged, or augmented transmission - are all at least qualitatively predictable given knowledge of the host species densities and competencies. Indeed, several studies have recently advanced the theoretical basis for understanding these complex interactions between species diversity, host abundance and competency, and pathogen transmission dynamics that likely result in variable disease outcomes in natural systems [4], [66].

### Greater tadpole infection at higher intraspecific density

As tadpole density increased, the total number of ETa parasites successfully encysted increased, as did the *per-capita* infection intensity (Figure 2, top row). The trend was similar for RO parasites, with nominally greater total cysts and slightly greater per-capita infection with higher tadpole densities, though we cannot be very confident in that pattern statistically. These results differ somewhat from previous findings. Johnson et al. [65] found greater total RO parasite numbers in treatments with higher density of *Bufo* tadpoles, but equivalent (and trending towards lower) *per-capita* infection intensity. Because there were no significant treatment effects on tadpole survival, mass, or parasite prevalence (proportion of hosts infected), the possible explanations for our results are narrowed. For example, Dare et al. [67] found *Rana pipiens* tadpoles were smaller, slower to metamorphose, and ultimately more heavily infected by the trematode *Manodistomum syntomentera* Stafford 1905 when raised at higher intraspecific densities. Similarly, Raffel et al. [68] found greater ETa infection in *Bufo* tadpoles at higher densities, and inferred that this was from reduced growth and longer maturation time due to resource competition, i.e. staying longer in the pond before metamorphosis meant greater exposure to parasites. We found similar patterns between intraspecific density and infection intensity, but without the possibility for the mechanisms these investigators posited, as none of our animals reached metamorphosis and escaped further parasitism.

We postulate several mechanisms that could explain increased per-capita infection of *Rana* tadpoles at higher density. One possibility is that higher host density cues the release of more cercariae from infected snails [32], [33]. This seems unlikely to us as infected snails were rotated among tanks on a daily basis, and thus were subject to cues from many different treatments. An additional mechanism which by itself would be insufficient to explain the pattern, but may have contributed, is that increasing host density might increase the per-capita success rate of parasites by reducing the ratio of parasites to hosts. Poulin [34] reported a non-linear, density-dependence in trematode parasite success rate, including for Echinostomes, among infection experiments; namely, that as parasite density (or “dose”) decreases, and the parasite to host ratio decreases, per-capita parasite success increases. This increased success could occur at lower per-capita cercarial doses because there are not enough parasites to induce anti-parasite behavior or a strong immune response, both of which are important amphibian responses that can limit trematode infection [69]–[71]. Physiological responses to increased host density itself might also result in similar effects on behavior and immune responses. However, given that we did not see density effects on tadpole survival or mass, two of the most common impacts of density in tadpoles, we think an effect of density on physiology is less likely in this case.

### Larger tadpoles had greater infection

The pattern of higher infection intensity by ETa in larger tadpoles (Figure 2) may have been caused by several mechanisms. First, larger individuals may be larger “targets” for actively-swimming and chemo-orienting trematode cercariae. Alternatively, individuals that are heavily parasitized may have increased mass due to pathological edema [62] resulting in water retention. Lastly, all tadpoles may have acquired cercariae at equal rates, but smaller individuals may have been more likely to die from the infection. The sharp delineation of intense infections above and below 0.37 g (see Figure 2) supports this last mechanism, and this size cut-off corresponds with differences in pathology from uniform experimental infections noted by Schotthoeffer et al. [37] among tadpoles of varying developmental stage.

### Interactions among trematode species

We found a negative relationship between infection intensity of ETa and RO at the level of individual tadpoles. Johnson and Buller [46] found a similar pattern in experimental infections – the addition of RO cercariae reduced ET metacercariae by ∼20% while the addition of ET reduced RO metacercariae by ∼30%. They posited that these reductions were mediated through host immune responses. A similar mechanism may have operated here, but we can’t rule out the possibility that the negative relationship was generated as an artifact of our experimental design: having either RO or ETa infected snails in a tank at any one time (though hosts experienced both throughout the experiment).

### Future directions

As we think about how our experimental results apply to natural systems, we must consider how density of these hosts might actually vary across gradients of host diversity. Given the nature of the ETa system and the particular host species, a modest degree of density substitution is likely as host diversity increases, as all three hosts feed on attached algae and detritus; other species of snails and tadpoles are known competitors [72], [73]. However, Bronmark et al. [74] showed that while tadpoles can negatively affect snail reproduction and growth, snails can facilitate tadpole growth and development. Kiesecker and Skelly [75] found gray tree frogs (*Hyla versicolor LeConte 1825*) chose to lay more eggs in artificial ponds without snails, and especially without snails infected with trematodes. Clearly then, how communities of these hosts assemble in terms of density may be very complex. Moreover, the interactions between more taxonomically and functionally disparate host species like those used here (tadpoles and snails) for ETa may be quite different than those among more functionally similar species of a single taxa, like the amphibian communities described by Johnson et al. [26].

Together, host diversity, density, and competence interact to determine community-wide parasite transmission; the relationship between species diversity and parasitism will depend on the traits of the particular species involved. Moving forward, the greatest progress will likely come from studies that determine under what conditions and with what community structure we see particularly strong or weak effects of diversity on parasite transmission, rather than attempts to confirm or contradict the dilution effect *per se*. Expanding the range of host-parasite systems for which we understand the relationship between community assembly order and host competence should be a research priority.

## Supporting Information

Table S1 **Summary of posterior probability distributions for NB GLMM models (2.5%, 50%, and 97.5% quantiles).** On the left are terms in the models, and on the right are custom treatment comparisons. Simplistically, a parameter or comparison for which the 2.5% to 97.5% quantiles don’t overlap zero is likely to be important.
(DOCX)

Table S2 **Statistical results for tadpole and snail survival, mass, and infection prevalence.** The factor called “Treatment” in models of snail responses refers to the comparison between the two treatments that contained that species, for instance RH and RHP for *Physa* snails.
(DOCX)

## References

[1] Dobson AP (2004) Population dynamics of pathogens with multiple host species. Am Nat 164: S64–S78. 15540143 10.1086/424681

[2] Rudolf VHW, Antonovics J (2005) Species coexistence and pathogens with frequency-dependent transmission. Am Nat 166: 112–118. 15937794 10.1086/430674

[3] Ostfeld RS, Keesing F (2012) Effects of host diversity on infectious disease. Annu Rev Ecol Evol Syst 43: 157–182 10.1146/annurev-ecolsys-102710-145022

[4] Mihaljevic J, Joseph M, Orlofske S, Paull S (2014) The scaling of host density and richness can change the direction, shape, and detectability of diversity-disease relationships. PLoS One 9: e97812. 24849581 10.1371/journal.pone.0097812 PMC4029764

[5] Hall SR, Becker CR, Simonis JL, Duffy MA, Tessier AJ, et al (2009) Friendly competition: evidence for a dilution effect among competitors in a planktonic host-parasite system. Ecology 90: 791–801. 19341148 10.1890/08-0838.1

[6] Belden LK, Wojdak JM (2011) The combined influence of trematode parasites and predatory salamanders on wood frog (*Rana sylvatica*) tadpoles. Oecologia 166: 1077–1086 10.1007/s00442-011-1946-8 21384178

[7] Mitchell CE, Tilman D, Groth JV (2002) Effects of grassland plant species diversity, abundance, and composition on foliar fungal disease. Ecology 83: 1713–1726.

[8] Johnson PTJ, Preston DL, Hoverman JT, Henderson JS, Paull SH, et al (2012) Species diversity reduces parasite infection through cross-generational effects on host abundance. Ecology 93: 56–64. 22486087 10.1890/11-0636.1

[9] Keesing F, Holt RD, Ostfeld RS (2006) Effects of species diversity on disease risk. Ecol Lett 9: 485–498 10.1111/j.1461-0248.2006.00885.x 16623733

[10] Rondelaud D, Vignoles P, Abrous M, Dreyfuss G (2001) The definitive and intermediate hosts of *Fasciola hepatica* in the natural watercress beds in central France. Parasitol Res 87: 475–478 10.1007/s004360100385 11411948

[11] Magnanou E, Fons R, Feliu C, Morand S (2006) Physiological responses of insular wild black rat (*Rattus rattus*) to natural infection by digenean trematode *Fasciola hepatica* . Parasitol Res 99: 97–101. 16470416 10.1007/s00436-005-0063-1

[12] Lichtenfels JR (1971) Morphological variation in the gullet nematode, *Gongylonema pulchrum* Molin, 1857, from eight species of definitive hosts with a consideration of *Gongylonema* from *Macaca* spp. J Parasitol 57: 348–355. 5553452

[13] Brunner JL, Duerr S, Keesing F, Killilea M, Vuong H, et al (2013) An experimental test of competition among mice, chipmunks, and squirrels in deciduous forest fragments. PLoS One 8: e66798 10.1371/journal.pone.0066798 23824654 PMC3688938

[14] LoGiudice K, Ostfeld RS, Schmidt KA, Keesing F (2003) The ecology of infectious disease: effects of host diversity and community composition on Lyme disease risk. Proc Natl Acad Sci U S A 100: 567–571 10.1073/pnas.0233733100 12525705 PMC141036

[15] Allan BF, Langerhans RB, Ryberg WA, Landesman WJ, Griffin NW, et al (2009) Ecological correlates of risk and incidence of West Nile virus in the United States. Oecologia 158: 699–708 10.1007/s00442-008-1169-9 18941794

[16] Joseph MB, Mihaljevic JR, Orlofske SA, Paull SH (2013) Does life history mediate changing disease risk when communities disassemble? Ecol Lett 16: 1405–1412 10.1111/ele.12180 24138175

[17] Martin LB, Weil ZM, Nelson RJ (2007) Immune defense and reproductive pace of life in *Peromyscus* mice. Ecology 88: 2516–2528. 18027755 10.1890/07-0060.1 PMC7204533

[18] Cronin JP, Welsh ME, Dekkers MG, Abercrombie ST, Mitchell CE (2010) Host physiological phenotype explains pathogen reservoir potential. Ecol Lett 13: 1221–1232 10.1111/j.1461-0248.2010.01513.x 20618842

[19] Palacios MG, Sparkman AM, Bronikowski AM (2011) Developmental plasticity of immune defence in two life-history ecotypes of the garter snake, *Thamnophis elegans*–a common-environment experiment. J Anim Ecol 80: 431–437. 21182520 10.1111/j.1365-2656.2010.01785.x

[20] Previtali MA, Ostfeld RS, Keesing F, Jolles AE, Hanselmann R, et al (2012) Relationship between pace of life and immune responses in wild rodents. Oikos 121: 1483–1492 10.1111/j.1600-0706.2012.020215.x

[21] Huang ZYX, de Boer WF, van Langevelde F, Olson V, Blackburn TM, et al (2013) Species’ life-history traits explain interspecific variation in reservoir competence: a possible mechanism underlying the dilution effect. PLoS One 8: e54341 10.1371/journal.pone.0054341 23365661 PMC3554779

[22] Huang ZYX, de Boer WF, van Langevelde F, Olson V, Blackburn TM, et al.. (2013) Correction: Species’ life-history traits explain interspecific variation in reservoir competence: A possible mechanism underlying the dilution effect. PLoS One 8. doi:10.1371/annotation/edc86621-7e9e-4702-b5d3-40cf19ebf731. 10.1371/journal.pone.0054341 PMC3554779 23365661

[23] Nunn CL (2002) A comparative study of leukocyte counts and disease risk in primates. Evolution (N Y) 56: 177–190. 10.1111/j.0014-3820.2002.tb00859.x 11913662

[24] Cooper N, Kamilar JM, Nunn CL (2012) Host longevity and parasite species richness in mammals. PLoS One 7: e42190 10.1371/journal.pone.0042190 22879916 PMC3413396

[25] Young H, Griffin RH, Wood CL, Nunn CL (2013) Does habitat disturbance increase infectious disease risk for primates? Ecol Lett 16: 656–663 10.1111/ele.12094 23448139

[26] Johnson PTJ, Preston DL, Hoverman JT, Richgels KLD (2013) Biodiversity decreases disease through predictable changes in host community competence. Nature 494: 230–233 10.1038/nature11883 23407539

[27] Lacroix C, Jolles A, Seabloom EW, Power AG, Mitchell CE, et al (2014) Non-random biodiversity loss underlies predictable increases in viral disease prevalence. J R Soc Interface 11: 20130947 10.1098/rsif.2013.0947 24352672 PMC3899862

[28] Civitello DJ, Pearsall S, Duffy MA, Hall SR (2013) Parasite consumption and host interference can inhibit disease spread in dense populations. Ecol Lett 16: 626–634 10.1111/ele.12089 23452184

[29] Karvonen A, Paukku S, Valtonen ET, Hudson PJ (2003) Transmission, infectivity and survival of *Diplostomum spathaceum* cercariae. Parasitology 127: 217–224. 12964824 10.1017/s0031182003003561

[30] Smith MJ, Telfer S, Kallio ER, Burthe S, Cook AR, et al (2009) Host-pathogen time series data in wildlife support a transmission function between density and frequency dependence. Proc Natl Acad Sci U S A 106: 7905–7909 10.1073/pnas.0809145106 19416827 PMC2672915

[31] Paller VG V, Kimura D, Uga S (2007) Infection dynamics of *Centrocestus armatus* cercariae (Digenea: Heterophyidae) to second intermediate fish hosts. J Parasitol 93: 436–439 10.1645/GE-997R.1 17539435

[32] Moné H, Théron A, Combes C (1986) Interaction between the *Biomphalaria glabrata* - *Schistosoma mansoni* host-parasite system and the non-target molluscs: influence on cercarial production. J Parasitol 72: 410–416. 3746562

[33] Mouritsen KN (2002) The *Hydrobia ulvae* - *Maritrema subdolum* association: influence of temperature, salinity, light, water-pressure and secondary host exudates on cercarial emergence and longevity. J Helminthol 76: 341–347 10.1079/JOH2002136 12498640

[34] Poulin R (2010) The selection of experimental doses and their importance for parasite success in metacercarial infection studies. Parasitology 137: 889–898 10.1017/S0031182009991624 20025825

[35] Johnson PTJ, Lunde KB, Ritchie EG, Launer AE (1999) The effect of trematode infection on amphibian limb development and survivorship. Science 284: 802–804. 10221912 10.1126/science.284.5415.802

[36] Johnson PTJ, Sutherland DR (2003) Amphibian deformities and *Ribeiroia* infection: an emerging helminthiasis. Trends Parasitol 19: 332–335. 12901930 10.1016/s1471-4922(03)00148-x

[37] Schotthoefer A, Cole RA, Beasley VR (2003) Relationship of tadpole stage to location of echinostome cercariae encystment and the consequences for tadpole survival. J Parasitol 89: 475–482 10.1645/0022-3395(2003)0890475:ROTSTL2.0.CO2 12880244

[38] Holland MP, Skelly DK, Kashgarian M, Bolden SR, Harrison LM, et al (2007) Echinostome infection in green frogs (*Rana clamitans*) is stage and age dependent. J Zool 271: 455–462 10.1111/j.1469-7998.2006.00229.x

[39] Rohr JR, Schotthoefer AM, Raffel TR, Carrick HJ, Halstead NT, et al (2008) Agrochemicals increase trematode infections in a declining amphibian species. Nature 455: 1235–1239 10.1038/nature07281 18972018

[40] Holland MP (2010) Echinostome-induced mortality varies across amphibian species in the field. J Parasitol 96: 851–855 10.1645/GE-2351.1 20469948

[41] Toldeo R, Fried B (2005) Echinostomes as experimental models for interactions between adult parasites and vertebrate hosts. Trends Parasitol 21: 251–254 10.1016/j.pt.2005.04.006 15922241

[42] Fried B, Toldeo R (2009) The biology of echinostomes: from the molecule to the community. New York, NY: Springer New York.

[43] Szuroczki D, Richardson JML (2009) The role of trematode parasites in larval anuran communities: an aquatic ecologist’s guide to the major players. Oecologia 161: 371–385 10.1007/s00442-009-1388-8 19543919

[44] Beaver P (1937) Experimental studies on *Echinostoma revolutum* (Froelich), a fluke from birds and mammals. Illinois Biol Monogr 15: 1–96.

[45] Detwiler JT, Bos DH, Minchella DJ (2010) Revealing the secret lives of cryptic species: Examining the phylogenetic relationships of echinostome parasites in North America. Mol Phylogenet Evol 55: 611–620 10.1016/j.ympev.2010.01.004 20064622

[46] Johnson PTJ, Buller ID (2011) Parasite competition hidden by correlated coinfection: using surveys and experiments to understand parasite interactions. Ecology 92: 535–541. 21608460 10.1890/10-0570.1

[47] Thieltges DW, Jensen KT, Poulin R (2008) The role of biotic factors in the transmission of free-living endohelminth stages. Parasitology 135: 407–426 10.1017/S0031182007000248 18208633

[48] Warren K, Peters P (1968) Cercariae of Schistosoma mansoni and plants – attempt to penetrate *Phaseolus vulgaris* and *Hedychium coronarium* produces a cercaricide. Nature 217: 647–648. 5637738 10.1038/217647a0

[49] Gosner KL (1960) A simplified table for staging anuran embryos and larvae with notes on identification. Herpetologica 16: 183–190.

[50] Crofton HD (1971) A quantitative approach to parasitism. Parasitology 62: 179–193.

[51] Lambert D (1992) Zero-inflated Poisson regression models with an application to defects in manufacturing. Technometrics 34: 1–14.

[52] Kéry M (2010) Introduction to WinBUGS for ecologists: A Bayesian approach to regression, ANOVA, mixed models, and related analyses. Burlington, MA: Academic Press. doi:10.1016/B978-0-12-378605-0.00054-5.

[53] Zuur AF, Saveliev AA, Ieno EN (2012) Zero inflated models and generalized linear mixed models. Highland Statistics, Ltd.

[54] Bavarri M, Berger J (2004) The interplay between Bayesian and frequentist analysis. Stat Sci 19: 58–80.

[55] Gelman A (1996) Inference and monitoring convergence. In: Wilks W, Richarson S, Spiegehalter D, editors. Markov chain Monte Carlo in practice. London: Chapman and Hall. 131–143.

[56] Wedderburn RVM (1974) Quasi-likelihood functions, generalized linear models, and the Gauss-Newton method. Biometrika 61: 439–447.

[57] R Development Core Team R (2011) R: A Language and Environment for Statistical Computing. R Found Stat Comput 1: 409 10.1007/978-3-540-74686-7

[58] Venables WN, Ripley BD (2002) Modern Applied Statistics with S. 4th ed. doi:10.2307/2685660.

[59] Fournier DA, Skaug HJ, Ancheta J, Ianelli J, Magnusson A, et al (2012) AD Model Builder: using automatic differentiation for statistical inference of highly parameterized complex nonlinear models. Optim Methods Softw 27: 233–249.

[60] Sturtz S, Ligges U, Gelman A (2005) R2WinBUGS: a package for running WinBUGS from R. J Stat Softw. 12: 1–16 10.1103/PhysRevLett.44.1404

[61] Spiegelhalter DJ, Thomas A, Best NG, Gilks WR (1996) BUGS: Bayesian inference Using Gibbs Sampling, Version 0.5, (version ii).

[62] Fried B, Pane PL, Reddy A (1997) Experimental infection of *Rana pipiens* tadpoles with *Echinostoma trivolvis* cercariae. Parasitol Res 83: 666–669. 9272555 10.1007/s004360050316

[63] Wojdak JM, Clay L, Moore S, Williams T, Belden LK (2013) *Echinostoma trivolvis* (Digenea: Echinostomatidae) second intermediate host preference matches host suitability. Parasitol Res 112: 799–805 10.1007/s00436-012-3203-4 23239089

[64] Van Buskirk J, Ostfeld RS (1995) Controlling Lyme Disease by modifying the density and species composition of tick hosts. Ecolological Appl 5: 1133–1140 10.2307/2269360

[65] Johnson PTJ, Hartson RB, Larson DJ, Sutherland DR (2008) Diversity and disease: community structure drives parasite transmission and host fitness. Ecol Lett 11: 1017–1026 10.1111/j.1461-0248.2008.01212.x 18616550

[66] Miller E, Huppert A (2013) The effects of host diversity on vector-borne disease: the conditions under which diversity will amplify or dilute the disease risk. PLoS One 8: e80279 10.1371/journal.pone.0080279 24303003 PMC3841118

[67] Dare OK, Rutherford PL, Forbes MR (2006) Rearing density and susceptibility of *Rana pipiens* metamorphs to cercariae of a digenetic trematode. J Parasitol 92: 543–547 10.1645/GE-674R1.1 16883998

[68] Raffel TR, Hoverman JT, Halstead NT, Michel PJ, Rohr JR (2010) Parasitism in a community context: trait-mediated interactions with competition and predation. Ecology 91: 1900–1907. 20715608 10.1890/09-1697.1

[69] Thiemann GW, Wassersug RJ (2000) Patterns and consequences of behavioural responses to predators and parasites in *Rana* tadpoles. Biol J Linn Soc 71: 513–528 10.1006/bijl.2000.0459

[70] Koprivnikar J, Forbes MR, Baker RL (2006) On the efficacy of antiparasite behaviour: a case study of tadpole susceptibility to cercariae of *Echinostoma trivolvis* . Can J Zool 84: 1623–1629.

[71] Daly EW, Johnson PTJ (2011) Beyond immunity: quantifying the effects of host anti-parasite behavior on parasite transmission. Oecologia 165: 1043–1050 10.1007/s00442-010-1778-y 20857146

[72] Osenberg CW (1989) Resource limitation, competition, and the influence of life history in a freshwater snail community. Oecologia 79: 512–519. 28313486 10.1007/BF00378669

[73] Holomuzki JR, Hemphill N (1996) Snail-tadpole interactions in streamside pools. Am Midl Nat 136: 315–327.

[74] Bronmark C, Rundle SD, Erlandsson A (1991) Interactions between fresh-water snails and tadpoles- competition and facilitation. Oecologia 87: 8–18. 28313346 10.1007/BF00323774

[75] Kiesecker JM, Skelly DK (2000) Choice of oviposition site by gray treefrogs: the role of potential parasitic infection. Ecology 81: 2939–2943.

