# Supplementary material for: Host Density and Competency Determine the Effects of Host Diversity on Trematode Parasite Infection
Source: PLoS One. 2014 Aug 13;9(8):e105059. doi: 10.1371/journal.pone.0105059 (PMC4132046; doi:10.1371/journal.pone.0105059)
Supplement: Table S1 — Summary of posterior probability distributions for NB GLMM models (2.5%, 50%, and 97.5% quantiles). On the left are terms in the models, and on the right are custom treatment comparisons. Simplistically, a parameter or comparison for which the 2.5% to 97.5% quantiles don’t overlap zero is likely to be important. (DOCX) [file pone.0105059.s001.docx]

|  |  |  |  |  |  |  |  |  |  |
| --- | --- | --- | --- | --- | --- | --- | --- | --- | --- |
|  | **Table S1.** Summary of posterior probability distributions for NB GLMM models (2.5%, 50%, and 97.5% quantiles). On the left are terms in the models, and on the right are custom treatment comparisons. Simplistically, a parameter or comparison for which the 2.5% to 97.5% quantiles don’t overlap zero is likely to be important. | | | | | | | | |
|  |  |  |  |  |  |  |  |  |  |
|  | **ET metacercariae in *Rana* tadpoles** | |  |  |  |  |  |  |  |
|  |  | **Quantiles** |  |  |  | **Quantiles** |  |  |  |
|  | **Term** | *2.5%* | *Median* | *97.5%* | **Comparison** | *2.5%* | *Median* | *97.5%* |  |
|  | Intercept | 2.069 | 3.15 | 4.327 | RP - R24 | -0.458 | 0.412 | 1.287 |  |
|  | log (Mass) | 0.649 | 0.996 | 1.369 | RH - R24 | -0.692 | 0.154 | 0.992 |  |
|  | Density | -0.011 | 0.027 | 0.061 | RP - R48 | -1.229 | -0.188 | 0.929 |  |
|  | *Physa* | -0.458 | 0.412 | 1.287 | RH - R48 | -1.490 | -0.47 | 0.645 |  |
|  | *Helisoma* | -0.692 | 0.154 | 0.992 | RHP - R48 | -2.327 | -1.267 | -0.14 |  |
|  | log(Mass) * Density | 0.009 | 0.021 | 0.033 | RHP - R16 | -1.432 | -0.464 | 0.494 |  |
|  | *Physa* * *Helisoma* | -2.401 | -1.030 | 0.3 | R16 - R24 | -0.467 | -0.2 | 0.103 |  |
|  | Tank | 0.659 | 0.859 | 1.158 | R16 - R48 | -1.867 | -0.799 | 0.412 |  |
|  | NB size parameter (*k*) | 1.398 | 1.591 | 1.808 | R24 - R48 | -1.400 | -0.599 | 0.309 |  |
|  |  |  |  |  |  |  |  |  |  |
|  | **RO metacercariae in *Rana* tadpoles** | |  |  |  |  |  |  |  |
|  |  | **Quantiles** |  |  |  | **Quantiles** |  |  |  |
|  | **Term** | *2.5%* | *Median* | *97.5%* | **Comparison** | *2.5%* | *Median* | *97.5%* |  |
|  | Intercept | -4.16 | -0.594 | 2.702 | RP - R24 | -3.788 | -0.585 | 2.403 |  |
|  | log (Mass) | -0.463 | 0.088 | 0.651 | RH - R24 | -3.024 | -0.015 | 2.903 |  |
|  | Density | -0.1 | 0.001 | 0.104 | RP - R48 | -3.914 | -0.462 | 2.689 |  |
|  | *Physa* | -3.788 | -0.585 | 2.403 | RH - R48 | -3.202 | 0.104 | 3.372 |  |
|  | *Helisoma* | -3.024 | -0.015 | 2.903 | RHP - R48 | -1.838 | 1.479 | 4.822 |  |
|  | ET | -0.013 | -0.008 | -0.003 | RHP - R16 | -1.859 | 1.330 | 4.533 |  |
|  | log(Mass) * Density | -0.012 | 0.006 | 0.024 | R16 - R24 | -0.776 | 0.050 | 0.840 |  |
|  | *Physa* * *Helisoma* | -2.453 | 1.924 | 6.757 | R16 - R48 | -3.106 | 0.199 | 3.361 |  |
|  | Tank | 1.952 | 2.702 | 3.938 | R24 - R48 | -2.329 | 0.149 | 2.521 |  |
|  | NB size parameter (*k*) | 0.849 | 1.124 | 1.929 |  |  |  |  |  |
|  | Zero-inflation parameter | <0.001 | 0.108 | 0.153 |  |  |  |  |  |
|  |  |  |  |  |  |  |  |  |  |
|  | **ET metacercariae in *Helisoma* snails** | |  |  |  |  |  |  |  |
|  |  | **Quantiles** |  |  |  |  |  |  |  |
|  | **Term** | *2.5%* | *Median* | *97.5%* |  |  |  |  |  |
|  | Intercept | -0.116 | 1.494 | 2.961 |  |  |  |  |  |
|  | log (Mass) | -1.787 | -0.279 | 1.263 |  |  |  |  |  |
|  | Treatment (RH vs. RHP) | -3.824 | -1.508 | 0.747 |  |  |  |  |  |
|  | log (Mass)* Treatment | -4.157 | -1.75 | 0.52 |  |  |  |  |  |
|  | Tank | 0.683 | 1.099 | 1.97 |  |  |  |  |  |
|  | NB size parameter (*k*) | 0.443 | 0.565 | 0.723 |  |  |  |  |  |
|  |  |  |  |  |  |  |  |  |  |
|  | **ET metacercariae in *Physa* snails** | |  |  |  |  |  |  |  |
|  |  | **Quantiles** |  |  |  |  |  |  |  |
|  | **Term** | *2.5%* | *Median* | *97.5%* |  |  |  |  |  |
|  | Intercept | 4.271 | 5.855 | 7.928 |  |  |  |  |  |
|  | log (Mass) | 0.648 | 1.266 | 2.024 |  |  |  |  |  |
|  | Treatment (RP vs. RHP) | -3 | -0.193 | 2.485 |  |  |  |  |  |
|  | log (Mass)* Treatment | -1.079 | -0.102 | 0.871 |  |  |  |  |  |
|  | Tank | 0.204 | 0.431 | 0.897 |  |  |  |  |  |
|  | NB size parameter (*k*) | 1.12 | 1.442 | 1.812 |  |  |  |  |  |
|  |  |  |  |  |  |  |  |  |  |
|  | **Total ET metacercariae in host communities** | | |  |  |  |  |  |  |
|  |  | **Quantiles** |  |  |  | **Quantiles** |  |  |  |
|  | **Term** | *2.5%* | *Median* | *97.5%* | **Comparison** | *2.5%* | *Median* | *97.5%* |  |
|  | Intercept | 3.952 | 4.768 | 5.615 | RP - R24 | 0.128 | 0.731 | 1.444 |  |
|  | Density | 0.017 | 0.042 | 0.070 | RH - R24 | -0.067 | 0.624 | 1.348 |  |
|  | *Physa* | 0.128 | 0.731 | 1.444 | RP - R48 | -1.102 | -0.277 | 0.592 |  |
|  | *Helisoma* | -0.067 | 0.624 | 1.348 | RH - R48 | -1.215 | -0.360 | 0.444 |  |
|  | *Physa* * *Helisoma* | -1.862 | -0.774 | 0.291 | RHP - R48 | -1.135 | -0.406 | 0.374 |  |
|  | NB size parameter (*k*) | 1.221 | 2.038 | 3.056 | RHP - R16 | 0.023 | 0.926 | 1.848 |  |
|  |  |  |  |  | R16 - R24 | -0.556 | -0.333 | -0.137 |  |
|  |  |  |  |  | R16 - R48 | -2.224 | -1.332 | -0.549 |  |
|  |  |  |  |  | R24 - R48 | -1.668 | -0.999 | -0.412 |  |
|  |  |  |  |  |  |  |  |  |  |
