# Supplementary material for: Host Density and Competency Determine the Effects of Host Diversity on Trematode Parasite Infection
Source: PLoS One. 2014 Aug 13;9(8):e105059. doi: 10.1371/journal.pone.0105059 (PMC4132046; doi:10.1371/journal.pone.0105059)
Supplement: Table S2 — Statistical results for tadpole and snail survival, mass, and infection prevalence. The factor called “Treatment” in models of snail responses refers to the comparison between the two treatments that contained that species, for instance RH and RHP for Physa snails. (DOCX) [file pone.0105059.s002.docx]

|  |  |  |  |  |  |
| --- | --- | --- | --- | --- | --- |
|  | **Table S2.** Statistical results for tadpole and snail survival, mass, and infection prevalence. The factor called "Treatment" for models for snail responses refers to the comparison between the two treatments that contained that species, for instance RH and RHP for *Physa* snails. | | | | |
|  |  |  |  |  |  |
|  |  |  |  |  |  |
|  | **Tadpole survival (binomial GLM)** | | |  |  |
|  |  | Estimate | SE | t | P |
|  | Intercept | 1.63 | 0.55 | 2.94 | 0.006 |
|  | density | -0.01 | 0.01 | -1.06 | 0.297 |
|  | H | 0.19 | 0.48 | 0.40 | 0.690 |
|  | P | -0.06 | 0.46 | -0.13 | 0.897 |
|  | H:P | -0.31 | 0.74 | -0.42 | 0.677 |
|  |  |  |  |  |  |
|  | **Tadpole mass (Gaussian GLMM)** | | |  |  |
|  |  | Estimate | SE | t | P |
|  | Intercept | -0.80 | 0.15 | -5.39 | <0.001 |
|  | density | -0.004 | 0.004 | -0.99 | 0.329 |
|  | H | 0.01 | 0.13 | 0.06 | 0.954 |
|  | P | -0.24 | 0.13 | -1.92 | 0.064 |
|  | H:P | 0.19 | 0.20 | 0.95 | 0.349 |
|  |  |  |  |  |  |
|  | **Prevalence of ET in tadpoles (binomial GLM)** | | | |  |
|  |  | Estimate | SE | t | P |
|  | Intercept | 2.11 | 0.76 | 2.76 | 0.010 |
|  | density | 0.01 | 0.02 | 0.26 | 0.796 |
|  | H | 0.13 | 0.65 | 0.20 | 0.846 |
|  | P | 0.31 | 0.70 | 0.45 | 0.658 |
|  | H:P | -0.53 | 1.06 | -0.50 | 0.621 |
|  |  |  |  |  |  |
|  | **Prevalence of RO in tadpoles (binomial GLM)** | | | |  |
|  |  | Estimate | SE | t | P |
|  | Intercept | -0.79 | 0.92 | -0.86 | 0.395 |
|  | density | 0.01 | 0.02 | 0.53 | 0.601 |
|  | H | 0.23 | 0.74 | 0.31 | 0.761 |
|  | P | -0.17 | 0.78 | -0.22 | 0.830 |
|  | H:P | 1.08 | 1.23 | 0.88 | 0.385 |
|  |  |  |  |  |  |
|  | ***Helisoma* mass (Gaussian GLMM)** | | |  |  |
|  |  | Estimate | SE | t | P |
|  | Intercept | 0.54 | 0.03 | 16.22 | <0.001 |
|  | Treatment | -0.01 | 0.05 | -0.30 | 0.769 |
|  |  |  |  |  |  |
|  | ***Physa* mass (Gaussian GLMM)** | | |  |  |
|  |  | Estimate | SE | t | P |
|  | Intercept | 0.077 | 0.005 | 14.241 | <0.001 |
|  | Treatment | -0.004 | 0.007 | -0.585 | 0.571 |
|  |  |  |  |  |  |
|  | ***Helisoma* survival (binomial GLM)** | | |  |  |
|  |  | Estimate | SE | t | P |
|  | Intercept | 2.97 | 0.59 | 5.03 | 0.001 |
|  | Treatment | -1.03 | 0.76 | -1.36 | 0.204 |
|  |  |  |  |  |  |
|  | ***Physa* survival (binomial GLM)** | | |  |  |
|  |  | Estimate | SE | t | P |
|  | Intercept | 1.16 | 0.32 | 3.59 | 0.005 |
|  | Treatment | 0.36 | 0.43 | 0.82 | 0.429 |
|  |  |  |  |  |  |
|  | **Prevalence of ET in *Helisoma* (binomial GLM)** | | | |  |
|  |  | Estimate | SE | z | P |
|  | Intercept | -0.37 | 0.13 | -2.74 | 0.006 |
|  | Treatment | -0.04 | 0.22 | -0.18 | 0.857 |
|  |  |  |  |  |  |
|  | **Prevalence of ET in *Physa* (binomial GLM)** | | | |  |
|  |  | Estimate | SE | z | P |
|  | Intercept | -0.06 | 0.17 | -0.34 | 0.737 |
|  | Treatment | 0.004 | 0.21 | 0.02 | 0.984 |
|  |  |  |  |  |  |
